# Mutational signatures of ionizing radiation in second malignancies

**DOI:** 10.1038/ncomms12605

**Published:** 2016-09-12

**Authors:** Sam Behjati, Gunes Gundem, David C. Wedge, Nicola D. Roberts, Patrick S. Tarpey, Susanna L. Cooke, Peter Van Loo, Ludmil B. Alexandrov, Manasa Ramakrishna, Helen Davies, Serena Nik-Zainal, Claire Hardy, Calli Latimer, Keiran M. Raine, Lucy Stebbings, Andy Menzies, David Jones, Rebecca Shepherd, Adam P. Butler, Jon W. Teague, Mette Jorgensen, Bhavisha Khatri, Nischalan Pillay, Adam Shlien, P. Andrew Futreal, Christophe Badie, ICGC Prostate Group, Colin S. Cooper, Rosalind A. Eeles, Douglas Easton, Christopher Foster, David E. Neal, Daniel S. Brewer, Freddie Hamdy, Yong-Jie Lu, Andrew G. Lynch, Charlie E. Massi, Anthony Ng, Hayley C. Whitaker, Yongwei Yu, Hongwei Zhang, Elizabeth Bancroft, Dan Berney, Niedzica Camacho, Cathy Corbishley, Tokhir Dadaev, Nening Dennis, Tim Dudderidge, Sandra Edwards, Cyril Fisher, Jilur Ghori, Vincent J. Gnanapragasam, Christopher Greenman, Steve Hawkins, Steven Hazell, Will Howat, Katalin Karaszi, Jonathan Kay, Zsofia Kote-Jarai, Barbara Kremeyer, Pardeep Kumar, Adam Lambert, Daniel Leongamornlert, Naomi Livni, Hayley Luxton, Lucy Matthews, Erik Mayer, Susan Merson, David Nicol, Christopher Ogden, Sarah O'Meara, Gill Pelvender, Nimish C. Shah, Simon Tavare, Sarah Thomas, Alan Thompson, Claire Verrill, Anne Warren, Jorge Zamora, Ultan McDermott, G. Steven Bova, Andrea L. Richardson, Adrienne M. Flanagan, Michael R. Stratton, Peter J. Campbell

**Affiliations:** 1 Cancer Genome Project, Wellcome Trust Sanger Institute, Wellcome Trust Genome Campus, Hinxton, Cambridgeshire CB10 1SA UK; 2 Department of Paediatrics, University of Cambridge, Cambridge CB2 0QQ, UK; 3 Oxford Big Data Institute and Oxford Centre for Cancer Gene Research, Wellcome Trust Centre for Human Genetics, Roosevelt Drive, Oxford OX3 7BN, UK; 4 The Francis Crick Institute, London WC2A 3LY, UK; 5 Department of Human Genetics, University of Leuven, Leuven B-3000, Belgium; 6 University College London Cancer Institute, Huntley Street, London WC1E 6BT, UK; 7 Histopathology, Royal National Orthopaedic Hospital NHS Trust, Stanmore, Middlesex HA7 4LP, UK; 8 Department of Paediatric Laboratory Medicine, The Hospital for Sick Children, Toronto, Ontario, Canada M5G 1X8; 9 Department of Genomic Medicine, MD Anderson Cancer Center, University of Texas, Houston, Texas 77030, USA; 10 Cancer Mechanisms and Biomarkers Group, Radiation Effects Department, Centre for Radiation Chemical and Environmental Hazards, Public Health England, Chilton, Didcot OX11 0RQ, UK; 11 Institute of Biosciences and Medical Technology, BioMediTech, University of Tampere and Fimlab Laboratories, Tampere University Hospital, Tampere FI-33520, Finland; 12 Dana-Farber Cancer Institute, Boston, Massachusetts 02215-5450, USA; 13 Brigham and Women's Hospital, Harvard Medical School, Boston, Massachusetts 02115 USA; 14 Department of Haematology, University of Cambridge, Hills Road, Cambridge CB2 2XY, UK; 15 Norwich Medical School and Department of Biological Sciences, University of East Anglia, Norwich NR4 7TJ, UK; 16 Division of Genetics and Epidemiology, The Institute Of Cancer Research, London SW7 3RP, UK; 17 Centre for Cancer Genetic Epidemiology, Department of Oncology, University of Cambridge, Cambridge CB1 8RN, UK; 18 University of Liverpool and HCA Pathology Laboratories, London WC1E 6JA, UK; 19 Urological Research Laboratory, Cancer Research UK Cambridge Institute, Cambridge CB2 0RE, UK; 20 Department of Surgical Oncology, University of Cambridge, Addenbrooke's Hospital, Cambridge CB2 0QQ, UK; 21 The Genome Analysis Centre, Norwich NR4 7UH, UK; 22 The University of Oxford, Oxford OX1 2JD, UK; 23 Department of Molecular Oncology, Barts Cancer Institute, Queen Mary University of London, John Vane Science Centre, London EC1M 6BQ, UK; 24 Statistics and Computational Biology Laboratory, Cancer Research UK Cambridge Institute, Cambridge CB2 0RE, UK; 25 The Chinese University of Hong Kong, Hong Kong, China; 26 Second Military Medical University, Shanghai 200433, China; 27 St George's Hospital, London SW17 0QT, UK; 28 Royal Marsden NHS Foundation Trust, London SW3 6JJ, UK; 29 School of Computing Sciences, University of East Anglia, Norwich NR4 7TJ, UK; 30 Cambridge University Hospitals NHS Foundation Trust, Cambridge CB2 0QQ, UK; 31 Oxford University Hospitals NHS Trust, John Radcliffe Hospital, Oxford OX3 9DU, UK.; 32 Statistics and Computational Biology Laboratory, Cancer Research UK Cambridge Institute, Cambridge CB2 0RE, UK

## Abstract

Ionizing radiation is a potent carcinogen, inducing cancer through DNA damage. The signatures of mutations arising in human tissues following *in vivo* exposure to ionizing radiation have not been documented. Here, we searched for signatures of ionizing radiation in 12 radiation-associated second malignancies of different tumour types. Two signatures of somatic mutation characterize ionizing radiation exposure irrespective of tumour type. Compared with 319 radiation-naive tumours, radiation-associated tumours carry a median extra 201 deletions genome-wide, sized 1–100 base pairs often with microhomology at the junction. Unlike deletions of radiation-naive tumours, these show no variation in density across the genome or correlation with sequence context, replication timing or chromatin structure. Furthermore, we observe a significant increase in balanced inversions in radiation-associated tumours. Both small deletions and inversions generate driver mutations. Thus, ionizing radiation generates distinctive mutational signatures that explain its carcinogenic potential.

## Abstract (web summary)

Ionizing radiation may induce irreparable DNA damage leading to cancer. Here, the authors identify a specific signature of mutations arising in patients exposed to ionizing radiation and suggest that radiation-induced tumorigenesis is associated with higher rates of genome-wide deletions and balanced inversions.

## Graphical abstract

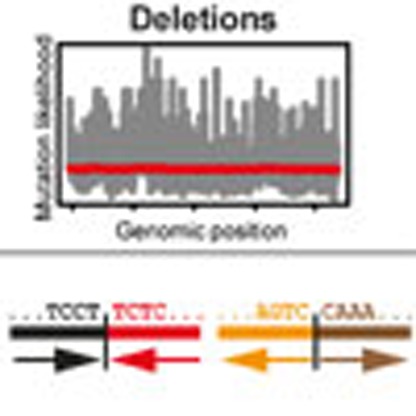

Exposure to ionizing radiation increases the risk of subsequent cancer. This risk exhibits a strong dose–response relationship, and there appear to be no safe limits for radiation exposure^1^. This association was first noted by March who observed an increased incidence of leukaemia amongst radiologists^2^. A leading cause of radiation-induced cancers appears to be exposure to medical radiation, either in the form of radiotherapy for an unrelated malignancy^3^ or diagnostic radiography^4^,^5^. These iatrogenic tumours arise as *de novo* neoplasms in a field of therapeutic radiation after a latency period that can span decades^6^, and are not recurrences of the original cancer^7^.

Many, but not all, environmental carcinogens induce cancer by increasing the rate of mutation in somatic cells. The physicochemical properties of a given carcinogen govern its interaction with DNA, leading to recurrent ‘signatures' or patterns of mutations in the genome. These can be reconstructed either from experimental model systems^8^,^9^ or from statistical analyses of cancer genomes in exposed patients^10^,^11^,^12^. Ionizing radiation directly damages DNA, and can generate lesions on single bases, single-stranded nicks in the DNA backbone, clustered lesions at several nearby sites and double-stranded DNA breaks^13^. In experimental systems exposed to radiation, including the murine germline and *Arabidopsis thaliana* cells, ionizing radiation can cause all classes of mutations, with possible enrichment of indels^14^,^15^,^16^,^17^,^18^,^19^,^20^,^21^,^22^. Targeted gene screens in radiation-induced sarcoma have indicated an increased burden of deletions and substitutions with frequent inactivation of *TP53* and *RB1* (refs 23, 24, 25). In addition, a transcriptome profile that represents a state of chronic oxidative stress has been proposed to be specific to radiation-associated sarcoma^26^.

We studied the genomes of 12 radiation-associated second malignancies of four different tumour types: osteosarcoma; spindle cell sarcoma; angiosarcoma; breast cancer. These were secondary tumours that arose within a field of therapeutic ionizing radiation and were not thought to be recurrences of the original malignancy treated with radiation. We chose this experimental design for several reasons: the tumours are classic radiotherapy-induced cancers with high attributable risks for the radiation exposure; the radiation exposure occurs over a short time period relative to the evolution of the cancer; and the mutational signatures of sporadic breast cancers and sarcomas have been well documented^10^,^27^,^28^,^29^. It should be noted that in the absence of biomarkers, a diagnosis of a tumour being radiation-induced cannot be definitively made (see Supplementary Note 1 for clinical details and further discussion).

We subjected these 12 tumours, along with normal tissues from the same patients, to whole-genome sequencing and obtained catalogues of somatic mutations. We compared our findings to 319 radiation-naive breast cancers and sarcomas processed by the same sequencing and bioinformatics pipeline: 251 breast tumours; 33 breast tumours with pathogenic *BRCA1* or *BRCA2* germline mutations; 35 osteosarcomas (see Methods for cohort details). In addition, we validated our findings in a published series of radiation-naïve and radiation-exposed prostate tumours from ten patients^30^.

The main aim of our analyses was to search for tumour-type independent, overarching signatures of ionizing radiation. Overall we identified two such signatures in radiation-associated second malignancies, an excess of balanced inversions and of small deletions.

## Results

### Tumour-type specific features

The 12 radiation-associated tumours harboured 1,506–9,245 substitutions per genome (median 4113), 135–943 indels per genome, (median 429) and 6–321 rearrangement break points per genome (median 74; Supplementary Data 1–3). The observed driver mutations followed the patterns expected for the tumour type consistently (Supplementary Table 1). Angiosarcomas harboured *PTPRB* and *PLCG1* mutations. In spindle cell sarcoma and osteosarcoma driver alteration of *TP53* and *CDKN2A* were found. Canonical *PIK3CA* mutations were seen in the radiation-associated breast tumours. Similarly, many of the mutational signatures seen in sporadic cancers were also present in radiation-associated tumours, such as chromothripsis in sarcomas (Supplementary Fig. 1). Against this backdrop of genomic diversity, we found evidence for two mutational signatures in the radiation-associated cancers that transcended tumour type: small deletions and balanced inversions.

### Enrichment of deletions in radiation-associated tumours

**Figure 1:**
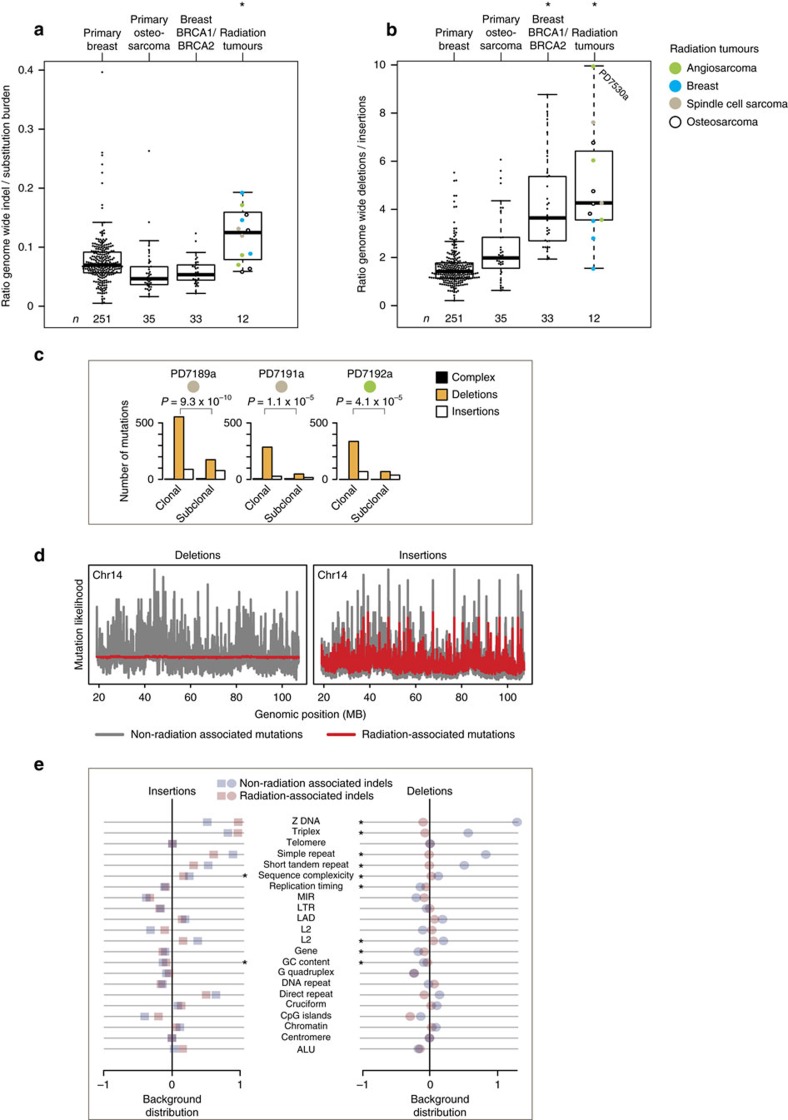
Indels in radiation-associated tumours. (**a**) Indel/substitution ratio. Shown is the indel/substitution ratio for each tumour. The ratio was significantly increased in radiation-associated second malignancies (0.0003, linear mixed effects model). Each dot represents a tumour. Different colours represent different tumour types (see legend, top right). Boxplots: vertical line – median; whiskers – minimum and maximum without outliers. (**b**) Deletion/insertion ratio. Shown is the deletion/insertion ratio of every tumour. Deletions were significantly (*) enriched in radiation-associated second malignancies (*P*<2.2 × 10^−16^, linear mixed effects model) and in breast tumours with germline *BRCA1* or *BRCA2* deficiency. Symbols, boxplots as per **a**. (**c**) Clonal versus subclonal indels in radiation-associated second malignancies. Shown are the absolute clonal (early) and subclonal (late) indel burdens of each tumour, by indel type. Amongst clonal indels, deletions were significantly enriched. *P*-values refer to the comparison of proportion of deletions/other indels in clonal versus subclonal indels (Fisher's exact test). (**d**) Indel likelihood across the genome. Shown is the probability of deletion or insertion to occur (vertical axis) across different regions of the genome (horizontal axis). The probability was modelled on the basis of associations between indels and genomic properties (see Methods). Chromosome 14 is shown as a representative chromosome. Radiation-associated indels were compared to indels of 35 non-radiation-associated osteosarcomas. Radiation-associated deletions, but not insertions, followed a more uniform distribution across the genome than in radiation-naive samples. (**e**) Distribution of indels in relation to genomic features. Comparison of the mutation density of radiation versus non-radiation indels in relation to genomic features. *X* axis: ratio of mutation density of non-radiation-associated indels or radiation-associated indels over background density. *Y* axis: genomic feature. The distribution of insertions in both radiation-associated and radiation-naïve tumours correlated with several genomic features, with few significant differences (asterisk) between the two. In contrast, the distribution of deletions in radiation-induced cancers, but not in radiation-naive tumours, showed little variability and resembled the background distribution more closely. Thus, significant differences (asterisk) were seen in the deletion density in relation to genomic features comparing radiation-associated and radiation-naïve tumours. *P*-values are detailed in Supplementary Data 4.

Although the absolute burden of indels varied across the 12 radiation-associated tumours, in each tumour the indel burden was high compared with that tumour's substitution burden (Fig. 1a). Compared with 319 radiation-naive tumours, the indel/substitution ratio was significantly increased in radiation-associated tumours (*P*=0.0003, linear mixed effects model, see Methods).

Deletions and insertions were not equally enriched. There was a significant excess of deletions relative to insertions in radiation-associated second malignancies (Fig. 1b; *P*<2.2 × 10^−16^, linear mixed effects model). This excess of deletions was also seen in *BRCA1* or *BRCA2* germline-deficient breast tumours, as previously described^27^, but was not seen in radiation-naive sporadic breast tumours or sarcomas. In each radiation-associated tumour, the radiotherapy had been given over a relatively short time period many years earlier. If the excess deletions we observed were directly attributable to ionizing radiation, then the enrichment should only be evident amongst the early, clonal mutations and not in the late, subclonal mutations. We were able to define subclones in three of the radiation-associated tumour genomes. Strikingly, compared with subclonal mutations, deletions were significantly increased compared with insertions amongst clonal mutations in all three cases (Fig. 1c, *P*<0.00005; Fisher's exact test).

### Distribution of deletions across the genome

Many mutational signatures show uneven distribution across the genome, especially those associated with carcinogens such as tobacco smoke and ultraviolet light^31^, thought to arise due to higher order chromatin organization and accessibility of the carcinogen and repair proteins to DNA (ref. 32). Deletions found in radiation-naive tumours showed considerable long-range variation in density across the genome, as did insertions in both radiation-associated and radiation-naive tumours, correlating with several genomic features (Fig. 1d,e; Supplementary Data 4). In stark contrast, deletions in radiation-induced cancers showed almost no variability across the genome and minimal correlation with genomic properties such as replication timing, sequence complexity or GC content. We hypothesize that this is because of the pervasive penetration of ionizing radiation through tissue, meaning that its interaction with DNA is stochastic and unaffected by higher order chromatin structure. Since small deletions are the predominant read-out of this damage, they show no association with the genomic features that influence other mutational processes.

### Evidence of non-homologous end-joining causing deletions

In two aspects, the deletions of radiation-associated cancers resembled those of *BRCA1* or *BRCA2* germline-deficient breast tumours (Supplementary Fig. 2): enrichment for deletions >2–3 bp in length and significantly higher rates of microhomology at the breakpoint junction (*P*=2 × 10^−16^, Kolmogorov–Smirnov test)^27^. This similarity suggests that microhomology mediated or non-homologous end-joining are the pathways for repairing radiation-induced DNA damage, rather than homologous recombination. Possible explanations for this include that damage occurs at phases of the cell cycle when homologous recombination pathways are less active^33^ or because the damaged DNA ends contain structures that interfere with homologous recombination^34^.

### Enrichment of balanced inversions

**Figure 2:**
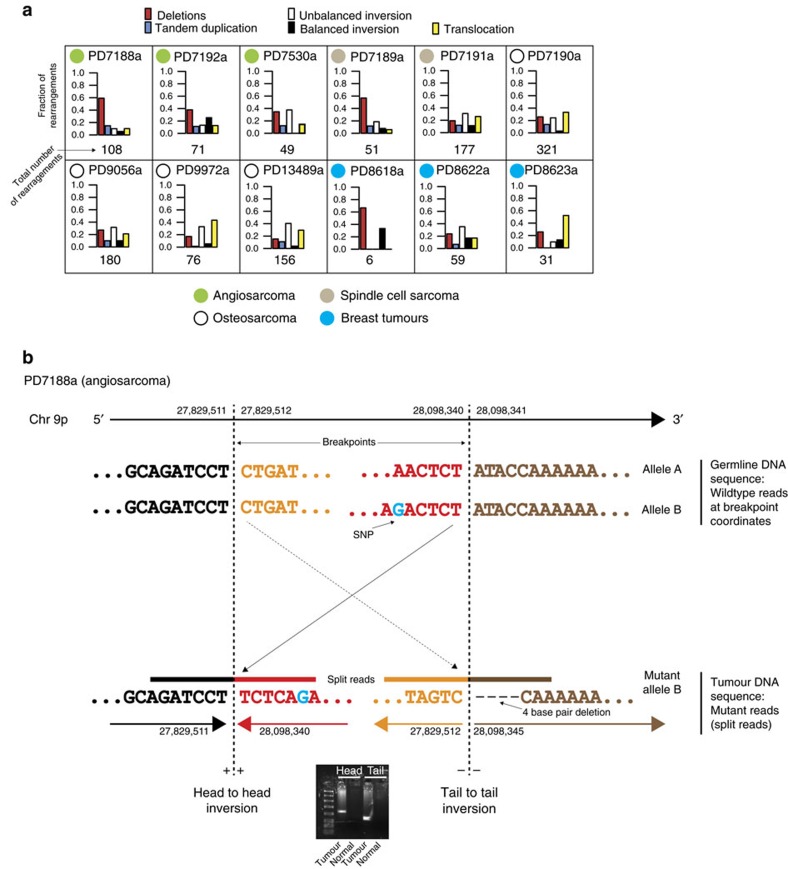
Balanced inversions in radiation-associated tumours. (**a**) Overview of rearrangements. Tumours exhibited tumour-type specific features. Balanced inversions (black bars) were found in every tumour, except PD7530a. (**b**) Example of a balanced inversion in PD7188a. A 0.9 Mb inversion. The inversion was validated by PCR across the breakpoint (gel image) and by split reads. Note that the split reads carried a heterozygous SNP at the head end.

For structural variants, we found enrichment of a rare type of rearrangement, balanced inversions, among radiation-associated second malignancies, irrespective of tumour type (Table 1; Fig. 2). While rearrangements with an inverted orientation are common in cancer genomes, they are typically unbalanced, associated with copy-number changes and caused by processes such as breakage-fusion-bridge cycles^35^, chromothripsis^29^ and chromoplexy^36^. We found a significant enrichment of balanced inversions in radiation-associated cancers: 52 in 11/12 tumours compared with 66 balanced inversions in 43 of the 286 radiation-naive tumours studied (*P*=2 × 10^−16^, generalized linear model). Of note, complete inversions were also significantly enriched amongst *BRCA1* and *BRCA2* germline-deficient breast tumours (Table 1, *P*=2 × 10^−16^). Balanced inversions ranged in size from a few hundred base pairs to nearly 100 megabases, and at breakpoint junctions showed variability in microhomology and in non-templated sequence inserted (Supplementary Data 5).

### Validation of findings in prostate tumours

**Figure 3:**
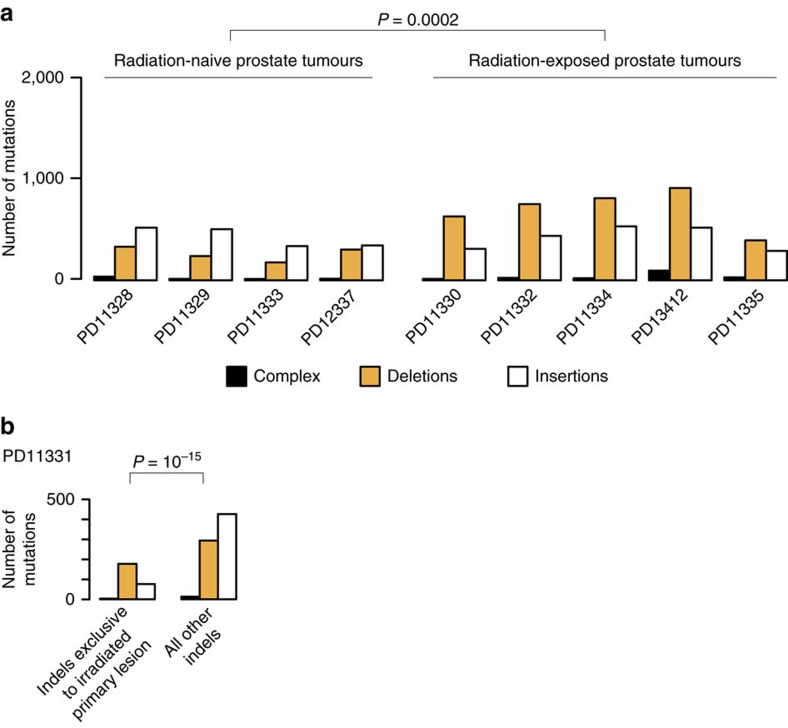
Indels in prostate tumours. (**a**) Indels in radiation-naive versus radiation-exposed prostate tumours. Shown is the indel burden, by indel subtype, found in radiation-naive and in radiation-exposed prostate tumours. In radiation-exposed tumours radiotherapy had been administered to the primary tumour before formation of metastases. Deletions were significantly enriched in radiation-exposed tumours (*P*=0.0002, generalized linear model). Note that radiation-associated tumours with confounding BRCA1 or BRCA2 deficiency were excluded from the statistical analysis (cases PD13412 and PD11335). (**b**) Indels in tumours from a patient whose primary lesion was treated with ionizing radiation after formation of metastases. Shown are indels that were found exclusively in the primary lesion and indels found in all other lesions. Deletions were significantly enriched amongst indels exclusive to the primary lesion. Comparison by Fisher's exact test, of the ratio of deletions over other indels.

To validate our observation that deletions and balanced inversions are genomic imprints of ionizing radiation, we examined the genomes of primary and/or metastatic prostate tumours from ten patients, previously published^30^. Five patients had developed metastases after irradiation of the primary lesion; four patients had never received radiation treatment; and one patient, PD11331, received radiotherapy to the primary lesion after metastases had already formed. Consistent with the observations made in the 12 radiation-associated second malignancies, we found a significant enrichment of deletions in prostate cancer lesions exposed to radiotherapy compared with radiation-naive tumours' (*P*=0.0002, generalized linear model, Fig. 3a). Strikingly, in patient PD11331, the radiation-exposed primary tumour (sample PD11331c), but not radiation-naive metastases, exhibited a preponderance of deletions (Fig. 3b, *P*=10^−15^, Fisher's exact test). Similarly, balanced inversions were enriched amongst radiation-exposed lesions (*P*=0.04, generalized linear model; Supplementary Table 2). In patient PD11331, again it was the radiation-exposed primary tumour, but not any of the metastases, that harboured a balanced inversion.

### Driver events generated by deletions and inversions

The oncogenic potential of a mutational process derives from its capacity to generate driver mutations. With their absence of copy-number effects, functional consequences of balanced inversions most commonly arise from genes broken at either end of the inversion, notwithstanding the possibility of long-range gene-enhancer disruption. In our data, 48/104 inversion break points disrupted or fused genes (Supplementary Data 5), with one forming a driver mutation through disruption of *TP53*. For the mutational signature of small deletions that we observe, we estimate that the median excess of indels in the radiation-induced cancers sequenced here is 201 indels per genome (linear mixed effects model, s.d. 348 indels). Among these are a 14 base pair deletion in *CASP8* and a 4 base pair deletion in *TP53*, both disrupting essential splice sites and thus generating driver events.

## Discussion

Overall we identified two genomic imprints of ionizing radiation, an excess of deletions and of an exceedingly rare type of rearrangement, balanced inversions. The validity of our study may be limited by the overall number of tumours we examined and the small number of each tumour type. Yet it would seem unlikely that the enrichment in radiation-associated tumours of deletions and of balanced inversions occurred by chance. This view is supported by our statistical analyses as well as the fact that the signatures were tumour-type independent. Both signatures were present across four different tumour types and could be validated in a cohort of radiation-exposed prostate cancer lesions, despite differences in the biological context of radiation-exposed prostate tumours and radiation-associated second malignancies (Supplementary Note 2). Particularly striking is patient PD11331 whose primary prostate lesion was irradiated after metastases had formed. The primary lesion, but not the metastases, exhibited the genomic features of ionizing radiation.

The relatively low number of mutations that we directly linked to ionizing radiation may seem surprising for such a well-known carcinogen. It is certainly considerably less than seen for cancers associated with tobacco, sunlight or aristolochic acid exposure^10^. This probably reflects the fact that although the attributable risk of such cancers is high, the absolute risk is relatively low. For example, >90% of angiosarcomas occurring after radiotherapy for primary breast cancer are attributable to radiation, but only one in a thousand women receiving such radiotherapy will develop angiosarcomas^37^, with a latency of many years. This suggests that although ionizing radiation clearly pushes bystander cells in the radiotherapy field towards cancer, the absolute burden of radiation-induced mutations per cell would not be high and additional driver mutations would be required.

## Methods

### Patient samples

Informed consent was obtained from all subjects and ethical approval obtained from Cambridgeshire 2 Research Ethics Service (reference 09/H0308/165). Collection and use of patient samples were approved by the appropriate institutional review board of each Institution.

### Whole-genome sequencing

DNA was extracted from 12 radiation-associated tumours and subjected to whole-genome sequencing, along with normal tissue derived from the same individuals. All tumour samples had been freshly frozen and were reviewed by reference pathologists. DNA extraction and preparation followed standard methods as previously described^38^. Reads were aligned to the reference human genome (NCBI37) by using BWA on default settings^39^. Reads which were unmapped or PCR-derived duplicates were excluded from the analysis. The average coverage of tumours was at least 40 × and of normal DNA 30 × , as per standard set by the International Cancer Genome Consortium.

### Variant detection

The CaVEMan (cancer variants through expectation maximization) algorithm was used to call single-nucleotide substitutions (github.com/cancerit/CaVEMan). To call insertions and deletions, we used split-read mapping implemented as a modification of the Pindel algorithm^38^. To call rearrangements we applied the BRASS (breakpoint via assembly) algorithm, which identifies rearrangements by grouping discordant read pairs that point to the same breakpoint event (github.com/cancerit/BRASS). Post-processing filters were applied to the output to improve specificity. Copy-number data were derived from whole-genome reads using the ASCAT (version 2.2) algorithm^40^. Mutations were annotated to Ensembl version 58.

### Variant validation

The precision of indels and substitutions presented here was assessed by manual inspection of 100 randomly selected substitutions and was found to be at least of 90% across the 12 radiation-associated tumours. This precision of coding indels and substitutions was confirmed by re-sequencing through whole-exome sequencing. Structural rearrangements were validated by defining exact break points through local reassembly, as implemented in BRASS. Only rearrangements that could be validated have been included in this report (listed individually in Supplementary Data 3).

### Screen/validation of balanced inversions

Rearrangement catalogues were screened for the presence of balanced inversions by means of a bespoke PERL script. Pairs of rearrangement calls were sought that were inversions in opposite directions with overlapping ranges of upper and lower break points. The search was directly performed on output from the Brass algorithm with the following post-processing filters: read count supporting the break point of greater than five reads and size of inversion greater than 2,500 base pairs unless the read count supporting the break point was greater than ten reads in which case no size threshold was applied. This post-processing strategy removes inversion artefacts, which are small and generally have a read count supporting the break point of less than five reads, without excluding small, high confidence inversions (defined as break points supported by at least ten reads). The precision of balanced inversion calls yielded by this search were assessed in the 12 radiation-associated tumours. In all but one balanced inversion, both rearrangements defining the inversion could be validated by algorithmic local reassembly or manual split-read mapping. In addition, a proportion of balanced inversions in 20/52 was subjected to PCR across the breakpoint in stock DNA from tumour and normal tissue. These inversions were all confirmed to be genuine and somatic (Supplementary Fig. 3).

### Germline variants

Germline point mutations in *TP53, BRCA1* and *BRCA2* were searched for in catalogues of germline indels and substitutions, as determined by the point mutation variant calling algorithms employed here. Putative mutations were compared against publicly available catalogues of pathogenic germline mutations in these genes (www.iarc.fr).

### Extraction of substitution signatures

Substitution signatures were extracted by using non-negative matrix factorization, as previously described^10^.

### Subclonality analyses

Subclonal tumour cell populations that exist within tumours can be screened for by searching for non-heterozygous mutations in mutation catalogues, as previously described, using a Dirichlet process^41^. This was applied to substitution and indel catalogues of the 12 radiation-associated whole genomes. However, with indels there is a concern that mutant read frequencies may be underestimated for larger indels, as reads containing larger indels may be less amenable to mapping. To overcome this bias, the indel mutant read frequencies were corrected by extracting unmapped reads (split reads) from sequencing reads. The Dirichlet process was then applied to indel catalogues with, and without, correction. The results in terms of number of subclonal peaks were indistinguishable whether corrected or uncorrected indel catalogues were analysed. Three of the twelve genomes screened for subclones contained subclones which corresponded to subclones defined by substitutions in these tumours. Thus, the indel-defined subclones were considered genuine. Indels were subdivided into clonal (peak of mutation copy number ∼1) or subclonal (peak of mutation copy number <1).

### Non-radiation tumours

A total of 319 tumours, 284 breast cancers and 35 osteosarcomas were included for comparison in analyses. These were spontaneous (primary), non-radiation-associated tumours. These tumours were sequenced to ∼40 × or more, along with normal tissue DNA from the same patients. These tumours were prepared, sequenced, analysed by the same pipeline as the 12 radiation-associated tumours, including use of the same algorithms. The osteosarcoma cases were a series of paediatric and adult tumours (sequencing data published in the European Genome-phenome Archive, accession EGAD00001000147). The breast tumours were comprised of oestrogen receptor positive and negative tumours^42^. For the purposes of this analysis they were subdivided into spontaneous cases (*n*=251) and those associated with pathogenic germline *BRCA1* or *BRCA2* mutation (*n*=33). No control primary angiosarcoma and spindle cell sarcomas were available for inclusion in our analyses.

### Association of mutation density with genomic features

The genomic properties listed in Supplementary Table 3 were calculated at every variant position, and, for comparison, at 100,000 random positions sampled uniformly from the callable regions of hg19. Only chromosomes 1–22 and X were considered. To test for differences in the genomic properties of variants in radiation-induced versus non-radiation-induced tumours, we used a two-proportion *z*-test for the binary variables, a *t*-test for the other quantitative variables (large sample size justifies central limit theorem), and a *χ*^2^-test for the categorical chromatin variable. A Benjamini-Yekutieli correction was applied to the raw *P*-values to account for multiple testing in the presence of likely correlation between these properties. Genomic properties are considered significantly different between radiation and non-radiation samples if the adjusted *q*-value is <0.01 and there is at least a 5% difference in magnitude between the two group means.

### Other statistical analyses

To assess whether radiation-associated tumours harbour significantly more indels relative to substitutions and more deletions relative to insertions, a mixed linear effects model was implemented using the R package lme4. After incorporating as fixed effects type of mutation (substitution, deletion and insertion) and group of tumour, interactions between tumour group and type of mutation were assessed. For comparison of indel size distribution underlying the clustering in Supplementary Fig. 2a, the statistic of the Kolmogorov–Smirnov test was used (command in R: ks.test(x,y)$statistic). Unless indicated, R was used for calculations.

### Data availability

Sequencing data have been deposited at the European Genome-Phenome Archive (EGA, http://www.ebi.ac.uk/ega/), which is hosted by the European Bioinformatics Institute; accession numbers EGAS00001000138; EGAS00001000147; EGAS00001000195.

## Additional information

**How to cite this article:** Behjati, S. *et al*. Mutational signatures of ionizing radiation in second malignancies. *Nat. Commun.* 7:12605 doi: 10.1038/ncomms12605 (2016).

## Supplementary Material

Supplementary Information Supplementary Figure 1.

Supplementary Data 1

Supplementary Data 2

Supplementary Data 3

Supplementary Data 4

Supplementary Data 5

## Figures and Tables

**Table 1:** Survey of balanced inversions in different tumour types.

	**Tumours with at least one balanced inversion**	**Number of tumours screened**	**Overall number of balanced inversions**
Primary breast tumours	39	251	59
Primary osteosarcoma	4	35	7
*BRCA1/2*-deficient breast tumours	19	33	46
Radiation-associated second malignancies	11	12	52

Rearrangement catalogues of tumours were searched informatically for the presence of balanced inversions. The basic principle of the search was to find pairs of head-to-head and tail-to-tail inversions in which the breakpoint coordinates overlap at both ends. Compared with all 286 primary tumours, balanced inversions were significantly enriched in radiation-associated second malignancies (*P*=2 × 10^−16^, generalized linear model) and also in *BRCA1* or *BRCA1* deficient breast tumours (*P*=2 × 10^−16^, generalized linear model). Further, compared to *BRCA1* or *BRCA1* deficient breast tumours balanced inversions were significantly enriched in radiation-associated second malignancies (*P*=0.0006, generalized linear model).
