# Supplementary material for: Mutational signatures of ionizing radiation in second malignancies
Source: Nat Commun. 2016 Sep 12;7:12605. doi: 10.1038/ncomms12605 (PMC5027243; doi:10.1038/ncomms12605)
Supplement: Supplementary Information — Supplementary Figure 1. [file ncomms12605-s1.pdf]

Supplementary Figure 1. Spectrum and signatures of substitutions.

a. Heatmaps of trinucleotide context of substitutions. Each square represents a substitution in a specific trinucleotide context, normalised against the prevalence of that trinucleotide in the human genome.
b. Spectrum of substitutions. Contribution (%) of the six substitution classes to overall substitution burden.
c. Contribution of signatures to individual tumours. Signatures nomenclature as per Alexandrov et al. (Nature 2013). Note that signature 30 is a novel signature, characterised by C>T mutations occuring mainly 5' of cyotinse or adenine (CpM). d. Genome wide substitution and indel burden.

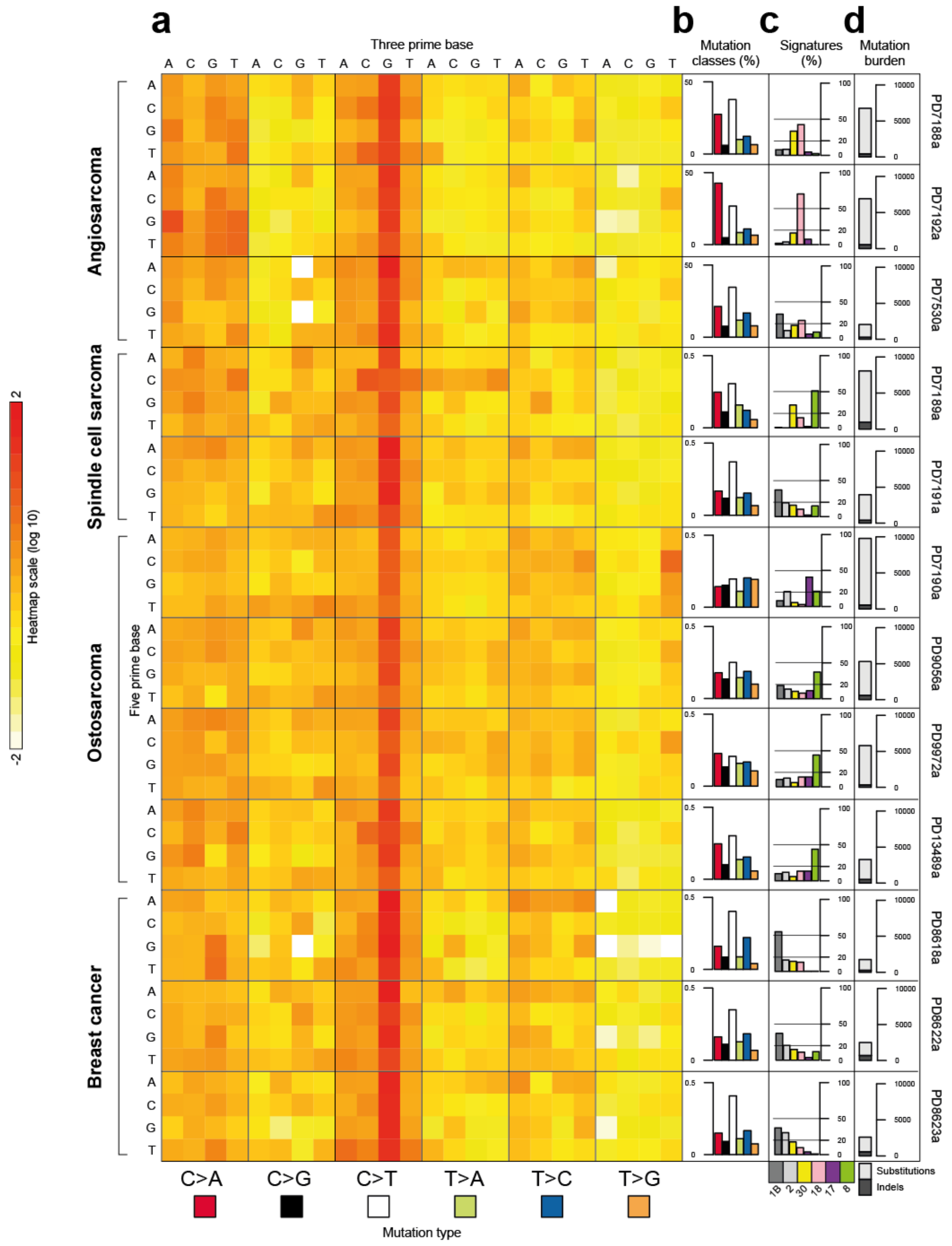

Supplementary Figure 2. Analyses of indel size, context, and motifs.

a. Indel size distribution in radiation-associated tumours. Clusters refer to results from a statistical comparison of the size distribution of radiation-associated tumours with non-radiation tumours including BRCA1 or BRCA2 germline deficient breast tumours (see methods).
b. Indel motifs. Motifs of indel sequences were analysed as described in methods. Shown here are the frequencies of indels with discernible motifs.
c. Comparison of deleted sequence with flanking 3' sequence. Left: Proportion of deletions that exhibit microhomology with 3' flanking sequence, or are repeats of the 3' flanking sequence, or neither. Right: Length of microhomology sequences in comparison to chance distribution. Differences are significant (p=2.2e-16, Kolmogorov Smirnov test).
d. One base pair indels that occur in homonymous mononucleotide repeat tracks.
e. Comparison of motifs in sequence on either side of indel. The frequency of each combination of motifs was measured. 0: no discernible motif; I: mononucleotide repeat; II: dinucleotide repeat; III: trinucleotide repeat; IV: tetranucleotide repeat.

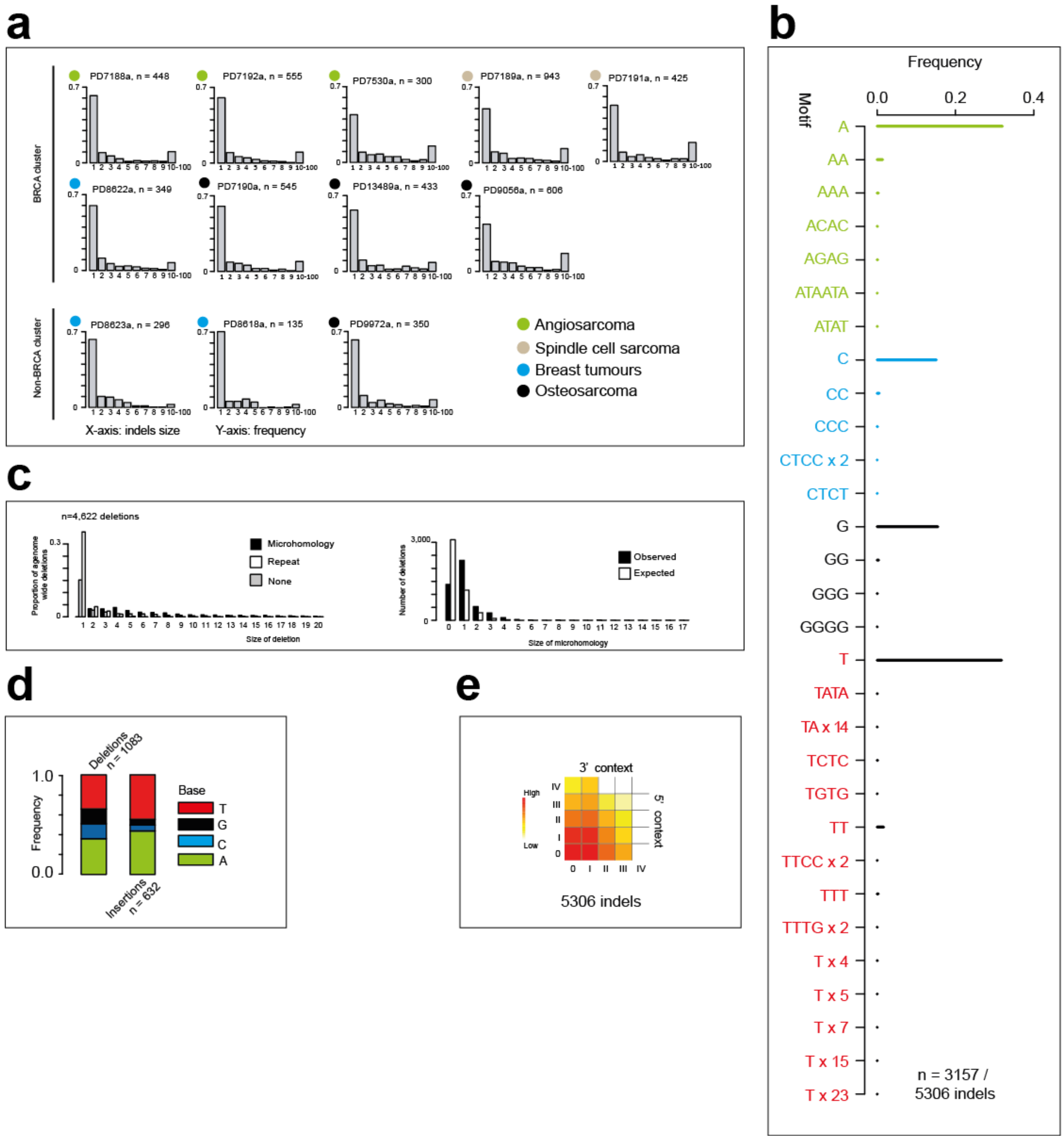

Supplementary Figure 3. PCR gels validating balanced inversions.

PCRs were performed in tumour and normal tissue DNA with primers placed on either side of the breakpoint of the rearrangement in question. T: Tumour DNA. N: Normal tissue DNA. Numbers refer to numbering of complete inversions as per Supplementary Table 6. Head: head end of inversion. Tail: tail end of inversion.

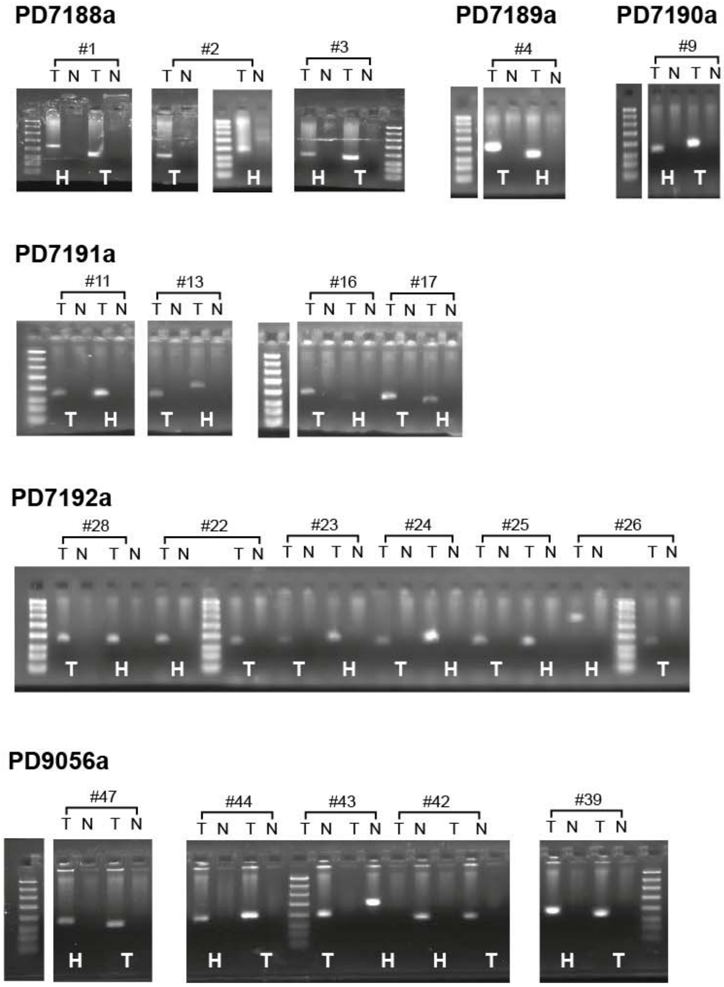

Supplementary Table 1. Overview of 12 radiation-associated tumours.

	Angiosarcoma			Spindle cell sarcoma		Osteosarcoma				Breast cancer			
**Tumour**	**PD7188a**	**PD7192a**	**PD7530a**	**PD7189a**	**PD7191a**	**PD7190a**	**PD9056a**	**PD9972a**	**PD13489a**	**PD8618a**	**PD8622a**	**PD8623a**	
Substitutions	6318	6362	1750	7147	3540	9245	4685	5459	2785	1506	1809	2020	
Kataegis*	-	-	-	-	Yes	Yes	Yes	-	-	-	-	-	
Indels	447	555	301	943	425	545	606	350	433	135	349	296	
Rearrangements	108	71	49	51	177	321	180	76	156	6	59	31	
Chromothripsis*	-	-	-	-	Yes	Yes	Yes	-	Yes	-	-	-	
**Driver mutations**													
TP53			red ▲		blue ■	blue ■■	red ✱	red ■	black ■				**Common sarcoma cancer genes**
CDKN2A				red ▶◀	red ▶◀								
PTPRB	red ●	blue ■ red ●											**Common angiosarcoma cancer genes**
PLCG1		blue ■											
MYC	blue ▲	blue ▲	blue ▲			blue ▲			blue ▲				
PIK3CA											blue ■	blue ■	**Common breast cancer genes**
CDH1										red ▲			
FOXA1											blue ■		
CHEK2	red ✚												**Others**
FGFR1						blue ▲							
KDM6A		red ✚											
NF1	red ✚												
CASP8		red ■											
ATRX						red ✚							
PTEN								red ▶◀					

red ▶◀ Homozygous deletion
red ✚ Deletion rearrangement#
red ✱ Inversion rearrangement
red ● Nonsense
red ▲ Essential splice site substitution
red ■ Essential splice site indel
blue ■ Missense
blue ▲ Amplification##
black ■ Germline missense

*Assessed visually
#Rearrangement deletions – deleted segments are < 1 Mb in size
##Amplicons – minimum number of copies: 9 in tetraploid genomes; 5 in diploid genomes

Supplementary Table 2. Prostate tumour series.

Case (ID used in this report)	ID in Gundem *et al.* (reference 5)	Type	Inversions	Substitution burden (truncal mutations)	Indel burden (truncal mutations)
PD11328	A10	No radiation	1	4555	894
PD11329	A22	No radiation	1	3795	764
PD11333	A12	No radiation	1	2562	532
PD12337	A21	No radiation	1	4277	669
PD11331	A31	Late	1*	2056	580
PD11330	A29	Radiation	3	5865	963
PD11332	A32	Radiation	6	4440	1224
PD11334	A24	Radiation	1	3256	1373
PD11335	A34	Radiation	4	3344	720
PD13412	A17	Radiation	0	9872	1537

*Inversion was found in primary tumour only and was absent from metastases

Supplementary Table 3: Genomic properties in hg19

Property	Note	Metric	Source	Ref
Centromere		Distance to (log_{10})	UCSC genome browser hg19 gap track	doi:10.1093/nar/gkt1168
Telomere	Same arm			
GC content	GC proportion of known nucleotides	Value in 100bp bins across genome	hg19 FASTA	
Trinucleotide complexity	Sum of 64 trinucleotide proportions squared. High value = low complexity.			
Chromatin state	E129 (osteoblasts) for osteosarcomas, consensus across all 127 cell types for prostate (no prostate in ROADMAP)	One of 25 chromatin segmentation states	ROADMAP	doi:10.1038/nature14248
Replication timing	Average across NHEK (normal skin), GM12878 (normal blood), IMR90 (normal lung). High value = early replicating	Average wavelet-smoothed signal in 1kb bins across genome	UCSC genome browser UW Repli-Seq tracks http://genome.ucsc.edu/cgi-bin/hgFileUi?db=hg19&g=wgEncodeUwRepliSeq	doi:10.1073/pnas.0912402107
Genes	Known protein-coding genes	Is position in or near (upto 100bp away) this feature? (Yes/No)	GENCODE v19	doi:10.1101/gr.135350.111
LADs	Tig3ET normal human embryonic lung fibroblasts		Lamina associated domains from Guelen et al.	doi:10.1038/nature06947
CpG islands	200+ bp with >50% GC content and more CG dinucleotides than expected (excluding repeat regions)		UCSC genome browser CpG islands track	doi:10.1093/nar/gkt1168
Direct repeats	10—300nt repeated directly one or more times 0—10nt away		Non-B DNA database v2	doi:10.1093/nar/gks955
G-quadruplexes	Four or more runs of three or more Gs, with 1—7nt spacers			
Cruciform inverted repeat proximity	Repeats of 6 or more nt repeated inversely up to 4nt away			
Triplex mirror repeat proximity	Repeats of 10 or more nt with 90% of one strand made of pyrimidines and repeated as a mirror up to 8nt away			
Short tandem repeat proximity	1—9nt repeated perfectly three or more times with no spacers			
Z-DNA motif proximity	Alternating purine-pyrimidine tracts of 10 or more nt, excluding AT/TA dinucleotide repeats			
ALU repeat			Repeatmasker 20140131 library	http://www.repeatmasker.org/genomes/hg19/RepeatMasker-rm405-db20140131/
MIR repeat				
L1 repeat				
L2 repeat				
LTR repeat				
DNA repeat				
Simple repeat	microsatellites			

Supplementary Note 1. Description and discussion of cases.

Clinical details of each of the twelve cases included in this experiment were provided by collaborating pathologists and are summarised in the table below. The cohort comprised four osteosarcomas, three angiosarcomas, three breast tumours and two spindle cell sarcomas. The latency period from primary tumour to secondary tumour ranged from 4 to 40 years (median 11 years). Note that one case, PD13489a, harboured a pathogenic germline mutation in *TP53.* The presence of pathogenic *TP53* germline mutations was excluded from the other cases by analysis of sequencing reads from normal tissue DNA (see Methods). In addition, we excluded the presence of pathogenic germline mutations in *BRCA1* and *BRCA2* from the study cohort, because there was some similarity between radiation-associated tumours and breast tumours arising in patients with *BRCA1* or *BRCA2* germline mutations.

Radiation-induced cancer has been defined as a malignancy which, following a latency period, forms in a field of radiation and is of a different tumour type than the original disease[1,2]. A key challenge in studying radiation-associated tumours is to substantiate the diagnosis with appropriate biomarkers. Thus, it is not possible to definitively ascertain whether the tumours we studied are radiation-associated. However, for the reasons discussed below, the cases we included can reasonably be regarded as radiation-associated second malignancies.

An alternative explanation for the presence of a common signature in four distinct cancer types could include a germline predisposing genotype. However, a common genotype would be unlikely to present across our diverse cohort of sarcomas and epithelial cancers (breast; prostate).

Angiosarcomas – PD7188a; PD7530a; PD7192a. Secondary angiosarcomas are defined by the presence of *MYC* amplification. All three cases under investigation harboured *MYC* amplification, and arose in a field of therapeutic radiation. Therefore, it is reasonable to consider these tumours to be radiation-associated. However, secondary angiosarcoma with *MYC* amplification can also be caused by chronic lymphoedema, which in turn can be caused by ionising radiation and by surgery. Thus, in cases of secondary angiosarcoma it is impossible distinguish with certainty between the effects of radiation, lymphoedema, and surgery.

Osteosarcomas - PD7190a; PD9056a; PD9972a; PD13489a. Osteosarcoma is a rare malignancy of bone. Development of osteosarcoma represents a well-known complication of radiotherapy, especially in patients with certain cancer-predisposing germline mutations. The four cases that we have studied can reasonably be regarded to be radiation-associated second tumours. However, one can speculate that PD7190a represents a recurrence of the original tumour, giant cell tumour of bone, as the two tumours are morphologically similar. However, this possibility can be excluded as PD7190a lacked the pathognomonic *H3F3A* G34W mutation of giant cell tumour of bone[5].

Radiations-associated second malignancies with clinical details

Note that all tumours arose in fields of ionising radiation.

ID	Sex	Age at diagnosis	Secondary tumour	Primary tumour	Latency (Yrs)	Comments
**PD7188a**	M	19	Angiosarcoma	Spindle cell sarcoma	7	Anatomical site > thigh
**PD7192a**	F	61	Angiosarcoma	Breast cancer	4	Patient underwent wide local excision
**PD7530a**	F	38	Angiosarcoma	Breast cancer	5	Patient underwent wide local excision with axillary clearance
**PD7190a**	M	25	Osteosarcoma	Giant cell tumour of bone	28	Osteosarcoma lacks *H3F3A* G34W mutation of giant cell tumour of bone
**PD9056a**	M	N/A	Osteosarcoma	Squamous cell carcinoma of tonsil	N/A	-
**PD9972a**	F	60	Osteosarcoma	Ovarian carcinoma	40	-
**PD13489a**	F	20	Osteosarcoma	Embryonal rhabdomyosarcoma	9	Patient has germline TP53 mutation
**PD7189a**	M	19	Spindle cell sarcoma	Synovial sarcoma	5	Spindle cell sarcoma lacks the t(18:X) translocation of synovial sarcoma
**PD7191a**	F	60	Spindle cell sarcoma	Breast cancer	16	Case has been reviewed to ascertain that it is not a sarcomatoid carcinoma
**PD8618a**	F	37	Breast cancer (hormone receptor positive)	Hodgkin's lymphoma	11	Patient received mantle radiotherapy
**PD8622a**	F	17	Breast cancer (hormone receptor positive)	Hodgkin's lymphoma	14	Patient received mantle radiotherapy
**PD8623a**	F	38	Breast cancer (hormone receptor positive)	Hodgkin's lymphoma	40	Patient received mantle radiotherapy

Spindle cell sarcomas – PD7189a; PD7191a. Primary spindle cell sarcoma is also an exceedingly rare tumour which decreases the odds of these tumours arising spontaneously in a field of ionising radiation. In case PD7189a, the primary and secondary tumour were synovial sarcoma and spindle cell sarcoma, respectively, which are related entities. The pathognomonic marker of synovial sarcoma[6], a t(18:X) translocation, was absent from the spindle cell sarcoma, PD7189a, indicating that this tumour is not a synovial sarcoma and thus represents a *de novo* secondary tumour. PD7191a arose in the field of radiation for a previous breast cancer. In rare cases, a breast carcinoma may be mistaken as a spindle cell sarcoma when morphologically it becomes sarcomatoid. We specifically requested expert review of this tumour which concluded that the appearance was typical of a spindle cell sarcoma and incompatible with a breast cancer.

Breast cancers - PD8618a, PD8622a, PD8623a. These case arose following mantle radiotherapy for Hodgkin's lymphoma. We chose these cases because mantle radiotherapy is a well-established cause of secondary breast cancer. In fact, it has been abandoned as a treatment of Hodgkin's disease. One could still argue that the tumours have arisen spontaneously, given the high incidence of spontaneous breast cancer. However, consideration of the attributable risk percentage (AR% = (relative risk – 1) / relative risk) suggests that most cases of breast cancer following radiotherapy for lymphoma can be attributed to radiotherapy (assuming a relative risk of 15-25 for developing breast cancer after radiotherapy for Hodgkin's disease[7]).

Supplementary Note 2. Comparison of radiation-exposed prostate tumours and radiation-associated second malignancies.

The biological context of radiation-associated second malignancies and radiation-exposed prostate lesions differs in two ways, although fundamental similarities remain.

First, the irradiated cells in prostate cancer are cancer cells as opposed to presumably normal cells in cases of second malignancies. The effects of ionising radiation may manifest differently in a cancer cell compared to a normal cell. However, one could also argue that the effects of ionising radiation on DNA are independent of whether the recipient cell is cancerous or normal. Our finding that deletions in radiation-associated tumours occur independent of sequence context and higher order chromatin structure would support this latter view.

Second, one may argue that the readout of the effects of ionising radiation in radiation-exposed tumours is more acute than in radiation-associated second malignancies. A latency period of many years may lie between irradiation and the development of a second tumour in a field of radiation. It is possible that during this period the genomic effects of ionising radiation are altered or become buried amongst new mutations. Our data that the deletion signature is stronger amongst early mutations compared to late mutations indicates that “burying” has taken place. However, importantly the signature remains apparent when looking at all mutations together, early and late.

Supplementary References

1. Cahan, W.G., Woodard, H.Q. & et al. Sarcoma arising in irradiated bone; report of 11 cases. *Cancer* **1**, 3-29 (1948).
2. Allan, J.M. & Travis, L.B. Mechanisms of therapy-related carcinogenesis. *Nat Rev Cancer* **5**, 943-55 (2005).
3. Guo, T. et al. Consistent MYC and FLT4 gene amplification in radiation-induced angiosarcoma but not in other radiation-associated atypical vascular lesions. *Genes Chromosomes Cancer* **50**, 25-33 (2011).
4. Kacker, C. et al. High frequency of MYC gene amplification is a common feature of radiation-induced sarcomas. Further results from EORTC STBSG TL 01/01. *Genes Chromosomes Cancer* **52**, 93-8 (2013).
5. Behjati, S. et al. Distinct H3F3A and H3F3B driver mutations define chondroblastoma and giant cell tumor of bone. *Nat Genet* **45**, 1479-1482 (2013).
6. Smith, S., Reeves, B.R., Wong, L. & Fisher, C. A consistent chromosome translocation in synovial sarcoma. *Cancer Genet Cytogenet* **26**, 179-80 (1987).
7. Horwich, A. & Swerdlow, A.J. Second primary breast cancer after Hodgkin's disease. *Br J Cancer* **90**, 294-8 (2004).
